# Subtype Classification and Prognosis Signature Construction of Osteosarcoma Based on Cellular Senescence-Related Genes

**DOI:** 10.1155/2022/4421952

**Published:** 2022-09-05

**Authors:** Hanyu Wang, Hongliang Liu, Li Wang, Shuchai Xu, Honglin Pi, Zhian Cheng

**Affiliations:** ^1^Department of Orthopedics, Xiangyang Hospital of Traditional Chinese Medicine, Hubei University of Chinese Medicine, Xiangyang, Hubei 441002, China; ^2^Department of Orthopedics, The Second Clinical Medical College, Guangzhou University of Chinese Medicine Guangdong Hospital of Chinese Medicine, Guangzhou University of Chinese Medicine, Guangzhou, Guangdong 510000, China

## Abstract

**Background:**

Cellular senescence (CS) is an alternative procedure that replaces or reinforces inadequate apoptotic responses and is used as an influencing factor for a variety of cancers. The value of CS gene in evaluating the immunotherapy response and clinical outcome of osteosarcoma (OS) has not been reported, and an accurate risk model based on CS gene has not been developed for OS patients.

**Methods:**

279 CS genes were obtained from CellAge. Univariate Cox regression analysis was used to screen the CS gene which was significantly related to the prognosis of OS samples in TARGET data set. The prognosis, clinicopathological features, immune infiltration, gene expression at immune checkpoints, tumor immune dysfunction and exclusion (TIDE) score, and chemotherapy resistance of OS were analyzed among clusters. Least absolute shrinkage and selection operator (Lasso) Cox regression analysis to build cellular senescence-related gene signature (CSRS). Univariate and multivariate Cox regression analysis of CSRS and clinical parameters were carried out, and the parameters with independent prognostic value were used to construct nomogram.

**Results:**

Based on 30 CS genes related to OS prognosis, OS samples were divided into three clusters: C1, C2, and C3. C3 showed the lowest survival rate and metastasis rate and the highest immune score and stromal score and was more likely to respond to immune checkpoint blockade (ICB) treatment. A CSRS scoring system including four CS genes (MYC, DLX2, EPHA3, and LIMK1) was constructed, which could distinguish the survival outcome, tumor microenvironment (TME) status, and ICB treatment response of patients with different CSRS score. Nomogram constructed by CSRS score and metastatic has a high prognostic value for OS.

**Conclusions:**

Our study identified a molecular classification determined by CS-related genes and developed a new CSRS that has potential value in OS immunotherapy response and clinical outcome prediction.

## 1. Introduction

Bone sarcoma is a rare component of malignant solid tumors, which are divided into a variety of histological types, including osteosarcoma (OS), chondrosarcoma, Ewing sarcoma, and chordoma which are the most common types of bone malignancies [1]. Distinct histological sarcomas have different clinical characteristics and results [2]. OS is one of the most common bone malignancies, accounting for 20% and 40% of all bone cancers, and is highly heterogeneous in terms of histological type, tumor site, and age of onset [2, 3]. Patients with local OS can achieve a 5-year survival rate of more than 70% through surgery combined with neoadjuvant and postoperative chemotherapy [4]. If recurrence or metastasis occurs, the 5-year survival rate of the patient is less than 20% [5]. Tumor grade is the best predictor of metastatic OS and survival at present [6]. A variety of systemic treatments have been explored for patients with recurrent or refractory OS, but the prognosis is not ideal. For most patients with metastatic OS, significantly improving the prognosis may require targeting a new process or molecule [5].

Cellular senescence (CS) is a mechanism that limits cell lifespan and constitutes a barrier to immortalization, which is characterized by stable cell cycle arrest triggered by stress [7]. Essentially, irreversible growth stagnation caused by cell senescence can inhibit precancerous cell proliferation. But, ironically, senescent cells can also create an immunosuppressive microenvironment that promotes malignant phenotypes and cancer progression [8]. CS has become an attractive concept of cancer treatment. The complete mechanism of apoptosis cannot be obtained in most identified malignant tumors. CS simulates programmed cell death by excluding cells from the active process of the cell cycle and becomes an alternative program to replace or enhance inadequate apoptotic responses [9]. Different CS phenotypes have been defined according to cell types and conditions, such as senescence induced by DNA damage, senescence induced by stress, and senescence induced by oncogenes [10]. Many genes play a role in CS. For example, CS releases a variety of secreted proteins, including inflammatory cytokines, chemokines, matrix remodeling factors, and growth factors, which play key but different roles in the tumor microenvironment [11]. With the advent of high-throughput technology, many CS genome resources and tools have been developed, such as SeneQuest [12], CellAge [13], and Human Cellular Senescence Gene Database (HCSGD) [14]. These resources and tools facilitate the study of CS in cancer gene signature. A study has constructed the CS gene signature by analyzing the CS gene in CellAge in patients with lung adenocarcinoma, which can be used as a potential index to evaluate the response to immunotherapy and prognosis of patients [15]. The value of CS gene in evaluating the response and clinical outcome of OS immunotherapy has not been reported, and an accurate CS-based risk model has not been developed for OS patients. Therefore, a definite CS gene signature should be developed in OS, but due to the complex, dynamic, and heterogeneous phenotype of senescent cells [16], it is also necessary to forcefully characterize specific CS subtypes.

In the current study, we aimed to classify the subtypes of OS by CS-related genes in OS patients and develop a new gene signature to evaluate the clinical outcome and immune microenvironment of OS patients, which may provide implications for the study of CS gene in cancer.

## 2. Materials and Methods

### 2.1. Source and Pretreatment of Osteosarcoma Sample Data and Cell Senescence-Related Genes

RNA-seq data and complete clinical features of OS were obtained from two databases, including Therapeutically Applicable Research to Generate Effective Treatments (TARGET, https://ocg.cancer.gov/programs/target) and Gene Expression Omnibus (GEO, https://www.ncbi.nlm.nih.gov/). The former dataset included 86 primary OS samples, and the sample data obtained from the latter database belonged to the GSE21257 data set, including 53 OS samples. CS-related gene motifs were obtained from CellAge (https://genomics.senescence.info/cells/) for a total of 279, of which 232 genes affect replicative CS, 34 genes affect stress-induced CS, and 28 genes affect oncogene-induced CS, and research process of this study is summarized as Figure S1. Consensus clustering analysis of OS samples is based on CS-related genes.

According to the expression of CS-related genes in TARGET, univariate Cox regression analysis with thresholds *P* < 0.05 was carried out by coxph function in *R*. The expression levels of CS-related genes screened by univariate Cox regression analysis were applied to consensus clustering analysis by ConsensusClusterPlus *R* package to identify new OS molecular subtypes. A total of 500 iterations were performed, each of which sampled 80% of the tumors. The *k* value that makes the cumulative distribution function (CDF) index close to the maximum value after multiple sampling was the best clustering number, in which the best *k* was selected from 2 to 9. The output results include CDF curve, Δ region diagram, and consensus matrix.

### 2.2. Clinicopathological Features in the Molecular Subtypes

To explore the clinical significance of molecular subtypes in OS, the relationship between molecular subtypes and prognosis and clinicopathological features of OS was studied. *R* packets “survival” and “survminer” were employed to generate Kaplan–Meier curves to evaluate the relationship between molecular subtypes and the prognosis of OS. Sample features include age, gender, and metastatic Huvos grade. The proportion of each clinical feature in different molecular subtypes was calculated and compared between molecular subtypes by log-rank test.

### 2.3. Assessment of Tumor-Infiltrating Immune Cells (TIICs) and Tumor Microenvironment (TME) Score in the Molecular Subtypes

CIBERSORT is an algorithm for characterizing the tissue composition of 22 human hematopoietic cell phenotypes, including seven T cell types, naïve and memory B cells, plasma cells, NK cells, and bone myeloid subsets [17]. This study evaluated the composition of immune cells of different subtypes in the two data sets using CIBERSORT. Estimation of Stromal and Immune cells in Malignant Tumors using Expression algorithm (ESTIMATE) [18] utilized expression data to analyze TME of malignant tumors. Through ESTIMATE, stromal score, immune score, and ESTIMATE score in OS TME were parsed here.

### 2.4. Function Enrichment Analysis within the Molecular Subtypes

To clarify the key process in which each molecular subtype is involved, Gene Set Enrichment Analysis (GSEA) was carried out using clusterProfiler package [19], and all candidate gene sets in Hallmark database [20] were used as background sets. False discovery rate (FDR) *q*-value <0.05 was considered to be statistically significant.

### 2.5. Expression of Immune Checkpoint and Drug Sensitivity in the Molecular Subtypes

The expression profiles of common immune checkpoints were downloaded and their expression differences among molecular subtypes were analyzed. The Tumor Immune Dysfunction and Exclusion (TIDE, https://tide.dfci.harvard.edu/) algorithm was implemented to compute TIDE score; lower TIDE score indicated a higher likelihood of response to immunotherapy. The half-maximal inhibitory concentration (IC50) of four chemotherapies in each OS subtype in TARGET database was estimated via Genomics of Drug Sensitivity in Cancer (GDSC; https://www.cancerrxgene.org/) using the *R* package “pRRophetic” [21].

### 2.6. Construction and Validation of Cellular Senescence-Related Gene Signature

The expression of 279 CS-related genes among OS molecular subtypes identified by consensus clustering analysis was analyzed, and the differentially expressed genes (DEGs) among subtypes were screened by Limma package. The overlapping genes of DEGs and prognostic CS-related genes screened by univariate Cox were used for further least absolute shrinkage and selection operator (Lasso) Cox regression analysis to construct cellular senescence-related gene signature (CSRS). The calculation formula of CSRS score was CSRS score = Σcoef (*i*) × exp (*i*). The *i* in the formula represents CS-related genes. According to CSRS, the CSRS score was calculated and normalized by *Z*-score in the samples of TARGET and GSE21257 data sets, and the generated Kaplan–Meier survival curve and receiver operating characteristic (ROC) curve were used as indicators to evaluate the effectiveness of CSRS.

### 2.7. Development and Evaluation of CS-Related Clinicopathological Nomogram

Univariate and multivariate Cox regression analysis was performed on CSRS and other clinical parameters (age, gender, and metastatic) using “survival” *R* package, and the parameters with independent prognostic value were obtained. *R* packet “rms” generated nomogram based on the results of independent prognostic analysis. The prediction accuracy and effectiveness of nomogram were evaluated by depicting the calibration curve, the decision curve analysis (DCA), and time-dependent ROC curves.

### 2.8. Statistical Analysis

All the statistical data of this study were analyzed by *R* software (version 4.0.2, https://www.r-project.org/). The Wilcoxon test assessed differences between the two groups of continuously distributed variables, and the Kruskal–Wallis test or chi-square test was used to compare data with normally and non-normally distributed variables. The comparison of each Kaplan–Meier curve was completed by the log-rank test. Time-dependent ROC curves were generated using the “survivalROC” package, consensus clustering analysis was performed by ConsensusClusterPlus *R* package. The difference analysis was carried out by Limma, and Lasso Cox regression was performed on “glmnet” *R* package. The results of immune infiltration assay were analyzed using ssGSEA, CIBERSORT *R* package, and ESTIMATE *R* package. The correlation matrix diagram was generated by Pearson correlation analysis. *P* < 0.05 was set as significant cutoff value.

## 3. Results

### 3.1. Three Molecular Subtypes Related to CS Were Identified in OS

**Figure 1:**
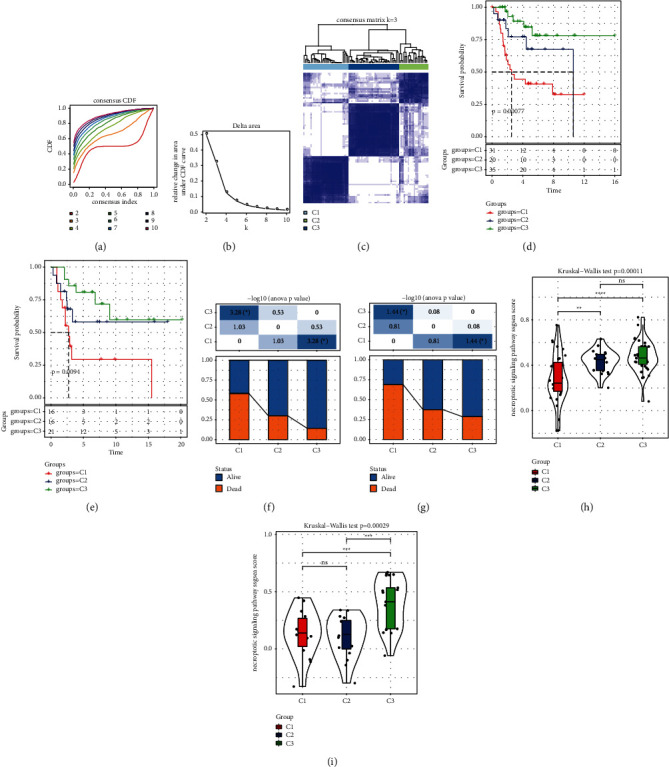
Establishment of molecular subtypes based on CS-related genes in OS: (a) CDF curve, each color represents a specific cluster. (b) Δ region diagram of consensus clustering. (c) Clustering heat map of samples at *k* = 3. (d) The trend of survival time of the three subtypes in TARGET. (e) The survival curve of the three subtypes in the GSE21257 dataset. (f) The survival status of the samples in the three subtypes of TARGET. (g) Survival and mortality ratios of the three subtypes in the GSE21257 dataset. (h) Necroptotic signaling pathway ssGSEA score in three subtypes of TARGET dataset. (i) Necroptotic signaling pathway score of three subtypes in GSE21257 dataset. ^*∗*^*P* < 0.05, ^*∗∗*^*P* < 0.01, ^*∗∗∗*^*P* < 0.001, ^*∗∗∗∗*^*P* < 0.0001, and ns means no significant difference.

Univariate Cox regression analysis of 279 CS-related genes showed that 30 genes were related to OS prognosis. 30 CS-related genes were inputted into ConsensusClusterPlus, and consensus clustering analysis was performed on the 86 OS samples according to the set parameters. When *k* = 3, the CDF curve gradually increases steadily, and when OS is divided into three subgroups, the clustering effect is the best and the stability within the group is better (Figures 1(a)–1(c)). Therefore, OS was classified into three molecular subtypes: C1, C2, and C3. There were significant differences in survival state and survival time among the three subtypes. The survival time of C3 was significantly longer than that of C1 and C2 (Figures 1(d) and 1(e)). In terms of survival status, the proportion of death samples in C3 was significantly lower than that in C1 and C2 (Figures 1(f) and 1(g)). The single sample GSEA (ssGSEA) score of the necroptotic signaling pathway in C3 was significantly higher than that in the other two subtypes (Figures 1(h) and 1(i)).

In addition, the clinical characteristics of the three molecular subtypes in the TARGET and GSE21257 data sets were compared, there was no significant difference in the distribution of present age and gender among the three subtypes of TARGET dataset. The proportion of metastatic patients belonging to C3 was significantly lower than that of the other two molecular subtypes (Figure S2A). Similar results were observed in the three molecular subtypes of the GSE21257 dataset, and there was no significant difference in the distribution of Huvos grade among the three subtypes in the dataset (Figure S2B).

### 3.2. TIICs and TME in Three CS-Related Subtypes

**Figure 2:**
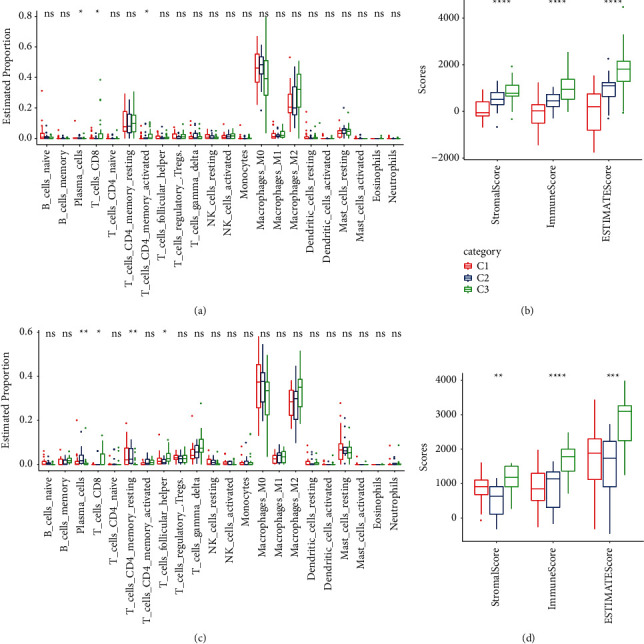
TIICs and TME in in three OS molecular subtypes: (a) TIICs proportion of three CS-related subtypes in TARGET. (b) Three TME scores of CS-related subtypes measured by ESTIMATE algorithm. (c) Proportion of 22 species of TIICs in three subtypes of GSE21257 dataset. (d) Stromal score, immune score, and ESTIMATE score in three OS molecular subtypes of GSE21257 dataset. Chi-square test, ^*∗*^*P* < 0.05, ^*∗∗*^*P* < 0.01, ^*∗∗∗*^*P* < 0.001, ^*∗∗∗∗*^*P* < 0.0001, and ns means no significant difference.

CIBERSORT was used to analyze the TIICs of three OS molecular subtypes. There were significant differences in plasma cells, CD8 T cells, and activated CD4 memory T cells estimated proportion of three CS-related subtypes in TARGET. The abundance of these three immune cells in C3 subtype was relatively higher than that of the other two molecular subtypes (Figure 2(a)). In the GSE21257 dataset, the proportion of plasma cells, CD8 T cells and helper follicular T cells, and resting CD4 memory T cells were significantly different among the three subtypes. The proportion of the first three cells was the highest in C3, and the proportion of the fourth cell in C1 and C2 was significantly higher than that in C3 (Figure 2(c)). The ESTIMATE algorithm calculated the TME score of three molecular subtypes in each OS dataset. The results indicated that C3 showed significantly higher stromal score and immune score and ESTIMATE score than the other two subtypes (Figures 2(b) and 2(d)). GSEA was also used to explore whether the three subtypes were associated with the immunomodulatory pathway of OS. Immune-related inflammatory response, interferon alpha response, interferon gamma response, and complement and allograft rejection in C1 were significantly suppressed relative to C3 (Figure S3A). In both OS datasets, the above immune-related pathways showed normalized enrichment scores (NESs) of less than 0 in C1 (Figure S3B).

### 3.3. Immunotherapy Response or Drug Sensitivity Assessment for Three CS-Related Subtypes

**Figure 3:**
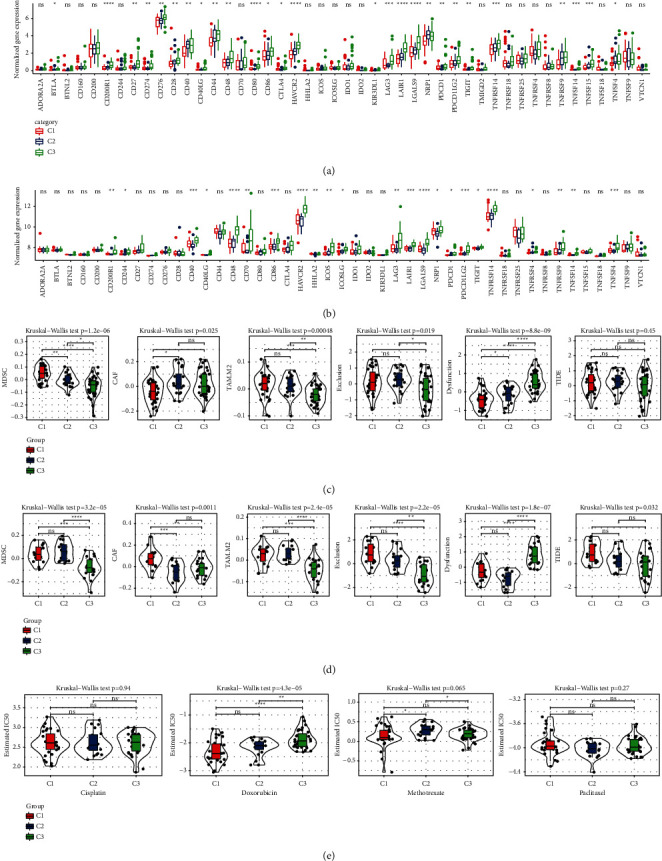
Immunotherapy response or drug sensitivity assessment for three CS-related subtypes: (a, b) the expression of immune checkpoints in three CS-related subtypes of TARGET and GSE21257. (c, d) Comparison of several scores calculated by TIDE in TARGET data and GSE21257 data sets among three OS molecular subtypes. (e) The IC50 of cisplatin, doxorubicin, methotrexate, and paclitaxel in three OS molecular subtypes.

Immune checkpoint blocking (ICB) is a commonly used immunotherapy. The expression analysis for several immune checkpoints [22] in three CS-related subtypes of two OS datasets showed that there were significant differences in the expression of a considerable number of immune checkpoints among the three subtypes (Figures 3(a) and 3(b)). TIDE algorithm was employed to evaluate the ICB responsiveness of three CS-related subtypes in two OS datasets. TIDE algorithm measured cancer-associated fibroblasts (CAFs) score, myeloid-derived suppressor cells (MDSCs) score, and M2 subtype of tumor-associated macrophages (TAMs) score, and two different mechanism scores of tumor immune escape, including the dysfunction score of tumor-infiltrating cytotoxic T lymphocytes (CTLs) (dysfunction) and the rejection score of CTLs by immunosuppressive factors (exclusion), as well as TIDE score for the molecular subtypes. For the three CS-related subtypes in TARGET, except for TIDE score, the other five scores showed significant differences among the three subtypes (Figure 3(c)). There were significant differences in all six scores of the three OS molecular subtypes in the GSE21257 dataset (Figure 3(d)). According to the performance of all these scores in the three OS subtypes, we speculated that C3 was more likely to respond to ICB treatment. The IC50 of four chemotherapeutic drugs for OS, cisplatin, doxorubicin, methotrexate, and paclitaxel, in three OS molecular subtypes was predicted. The sensitivity of the three CS-related subtypes to doxorubicin was significantly different. C1 was significantly associated with doxorubicin drug sensitivity, and C3 was significantly associated with doxorubicin drug resistance (Figure 3(e)).

We also explored the immune response or drug sensitivity of three CS-related subtypes in another osteosarcoma dataset, GSE39055. In this data set, there were also significant differences in survival rate among three CS-related subtypes. The survival rate order of subtypes was the same as that of subtypes in the other two data sets (Figure S4A). The symbolic immune checkpoints are associated with ICB therapy, including CD274 (PD-L1), CTLA4, and PDCD1(PD-1), with significant differences in expression among the three subtypes (Figure S4B). The responses of three CS-related subtypes in GSE39055 to cisplatin, doxorubicin, methotrexate, and paclitaxel were evaluated. There was a significant difference of three CS-related subtypes of estimated IC50 for doxorubicin. The C1 that adopted chemotherapy seemed to express more sensitivity (Figure S4C). Construction and verification of CSRS.

**Figure 4:**
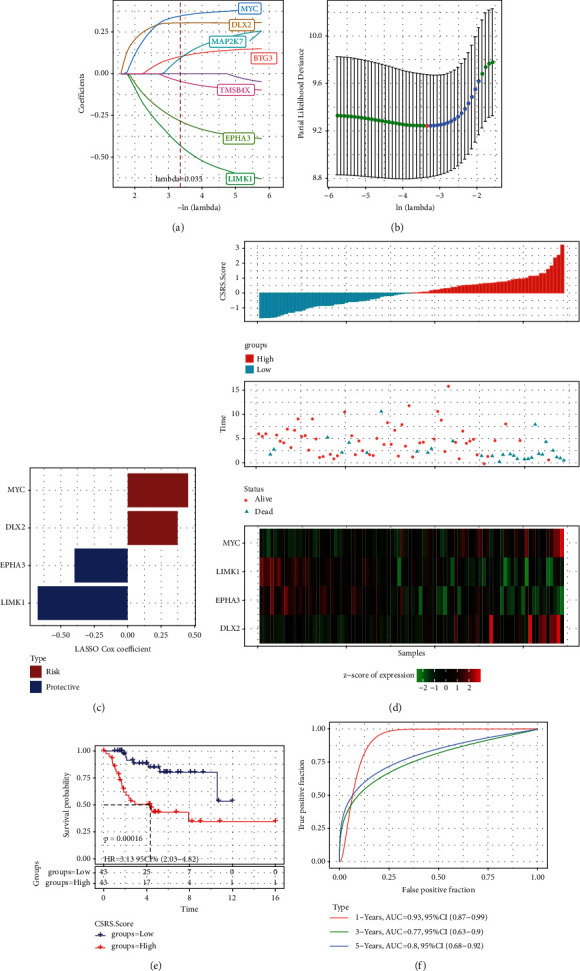
Construction of CSRS in TARGET: (a) the Lasso coefficient spectra of 8 CS-related genes. (b) The relationship between partial likelihood deviance and log (*λ*) in Lasso. (c) The Lasso Cox coefficient of the gene in CSRS, red represents the positive coefficient of the gene and blue represents the negative coefficient of the gene. (d) Normalized CSRS score, survival status, and expression heat map of four CS-related genes in all OS samples of TARGET. (e) Survival time and survival rate of two risk groups in TARGET. (f) The time-dependent ROC curve of CSRS.

Through the intersection of 30 prognostic CS-related genes and DEGs among molecular subtypes, 8 genes were obtained. 7 of the 8 genes were identified by Lasso analysis (Figures 4(a) and 4(b)). The multivariable stepwise Cox PHR analysis selected four genes to construct CSRS, including two risk genes (MYC and DLX2) and two protective genes (EPHA3 and LIMK1) (Figure 4(c)). CSRS endowed each sample with CSRS score and normalized them with Z-score, sorted according to the normalized CSRS score of all OS samples in TARGET, and generated the survival state map of the sample and the expression heat map of four CS-related genes. We observed from the figure that the higher the CSRS, the greater the likelihood of death. The expression of two risk genes increased with the increase of CSRS score, while the expression of two protective genes decreased with the increase of CSRS score (Figure 4(d)). All normalized CSRS scores in TARGET were divided into two groups with a critical value of 0. Patients with normalized CSRS score >0 were classified as the “high-risk group,” otherwise, as the “low risk group.” CSRS score could clearly distinguish between survival time and survival rate of samples in TARGET (Figure 4(e)). The area under the ROC curve for 1-year, 3-year, and 5-year survival rates was 0.93, 0.77, and 0.8, respectively (Figure 4(f)). CSRS score could also clearly distinguish the survival outcomes of patients with different risks in GSE21257 datasets (Figure S5A). The AUC of 1-year, 3-year, and 5-year survival were 0.74, 0.83, and 0.72, respectively (Figure S5B). Therefore, the accuracy of CSRS in predicting the survival result of OS is good.

### 3.4. Evaluation of the Effectiveness of CSRS in Clinical Application

**Figure 5:**
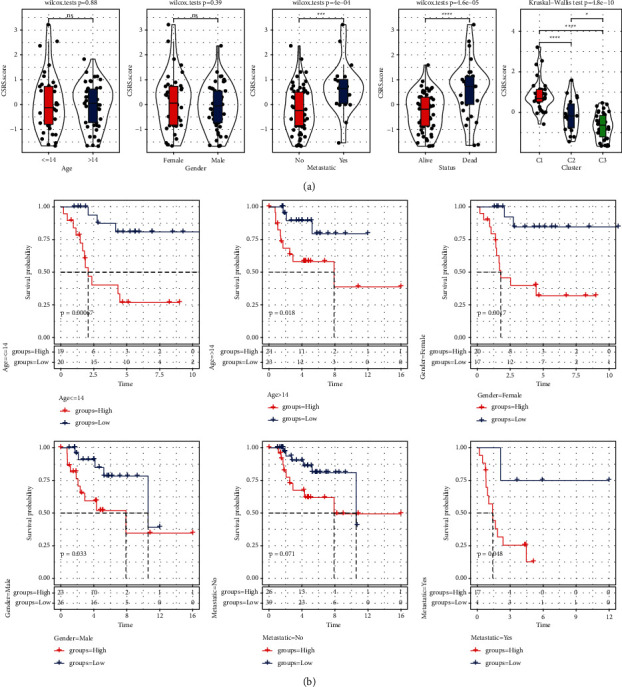
Evaluation of the effectiveness of CSRS in clinical application: (a) CSRS score of different clinicopathological parameters in TARGET dataset. (b) Survival curves for subgroups stratified by clinical characteristics.

We drew violin diagrams with Wilcoxon test and Kruskal test to compare CSRS score with different clinicopathological parameters in TARGET dataset. CSRS scores differed significantly within age, gender, TERM, and CS-related molecular subtype groups (Figure 5(a)). To further clarify the effectiveness of CSRS in clinical application, stratified survival analysis was carried out. The results showed that CSRS score maintained its predictive ability in all clinical subgroups (age ≤ 14, age > 14, male, female, and no metastatic). The survival rate of high CSRS score was significantly lower than that of low CSRS (Figure 5(b)).

### 3.5. Evaluation of TIICs, TME Score of CSRS, and the Correlation between CSRS and KEGG Signaling Pathway

**Figure 6:**
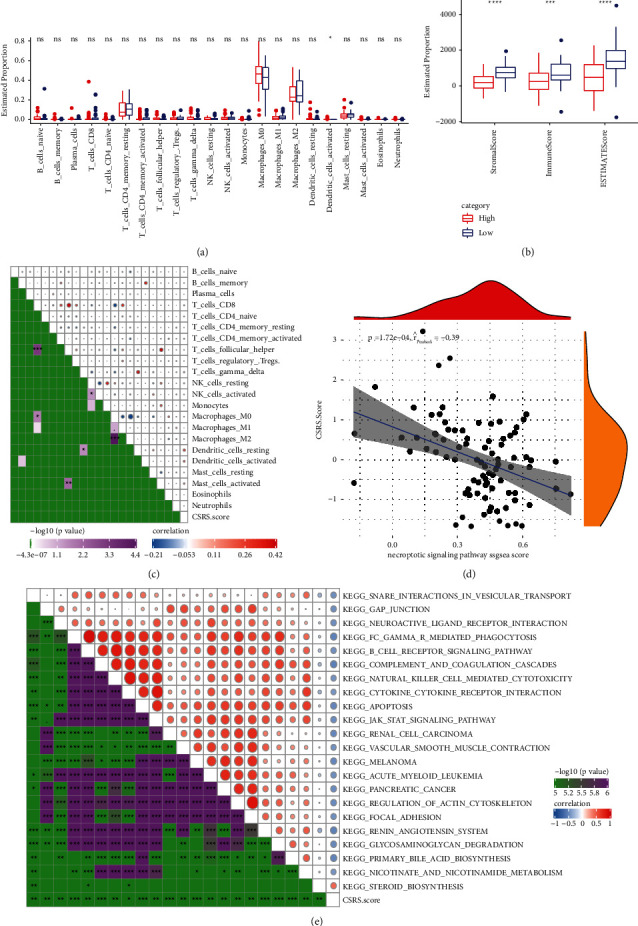
Evaluation of TIICs, TME score of CSRS, and the correlation between CSRS and KEGG signaling pathway: (a) estimated proportion of 22 TIICs based on CSRS score in TARGET. (b) The correlation matrix between CSRS and 22 TIICs in CIBERSORT. (c) Pearson correlation analysis of CSRS and necroptotic signaling pathway. (d) Correlation analysis between KEGG pathway and CSRS score with correlation greater than 0.4.

The estimated proportion of 22 TIICs based on CSRS score showed that 21 TIICs did not show significant difference in estimated proportion between the two CSRS score groups (Figure 6(a)). Correlation analysis also found no significant correlation between CSRS score and these TIICs (Figure 6(b)). The stromal score, immune score, and ESTIMATE score determined by ESTIMATE were related to the lower CSRS score (Figure 6(c)). The correlation analysis of CSRS and necroptotic signaling pathway showed that there was a significant negative correlation between them (Figure 6(d)). SsGSEA based on OS samples in TARGET dataset showed that CSRS score had a significant strong correlation with all KEGG paths in the matrix graph (Figure 6(e)).

### 3.6. Determination of Immunotherapy and Chemotherapeutic Drug Response Based on CSRS Score

**Figure 7:**
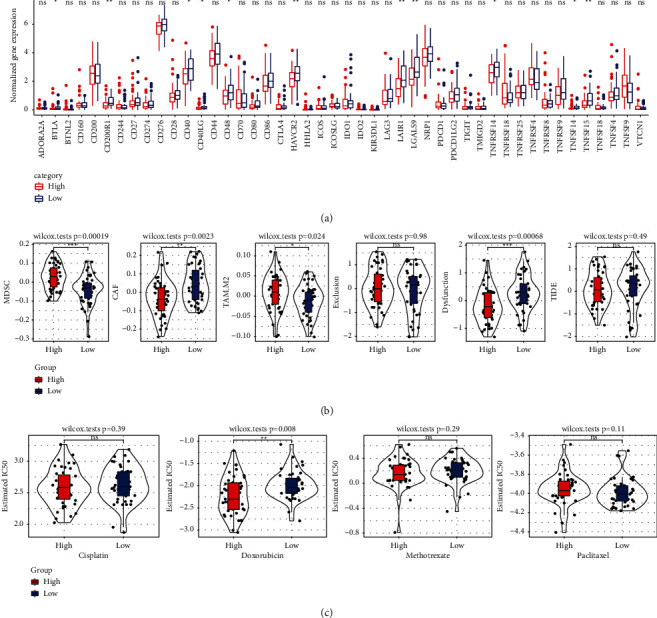
Determination of immunotherapy and chemotherapeutic drug response based on CSRS score: (a) analysis of immune checkpoint difference between two CSRS score groups in TARGET. (b) Six score differences calculated by TIDE based on CSRS score. (c) Sensitivity analysis of cisplatin, doxorubicin, methotrexate, and paclitaxel based on CSRS score.

Based on CSRS score, we compared the immune checkpoint expression between the two risk groups. Among all the immune checkpoints examined [22], the expression of BTLA, CD200R1, CD40, CD40LG, HAVCR2, LAIR1, LGALS9, TNFRSF14, TNFSF14, and TNFSF15 in low CSRS score group was significantly higher than that in high CSRS score group (Figure 7(a)). The MDSC score and M2.TAM score of high CSRS score group were significantly higher than those of low CSRS score group, and the CAF score and dysfunction of high CSRS score group were significantly lower than those of low CSRS score group (Figure 7(b)), and CSRS score could also significantly distinguish the degree of response of OS patients to doxorubicin (Figure 7(c)).

### 3.7. Development and Evaluation of Nomogram Combined CSRS Score and Clinicopathological Features Associated with OS Prognosis

**Figure 8:**
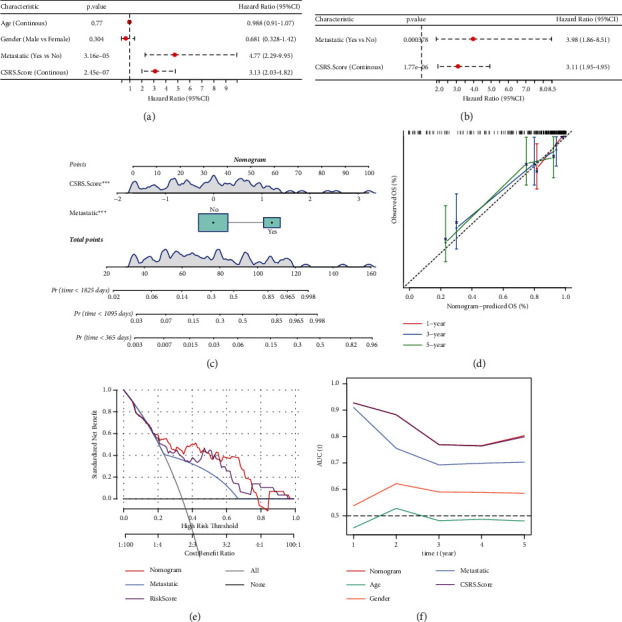
Development and evaluation of nomogram combined CSRS score and clinicopathological features associated with OS prognosis: (a) univariate Cox regression analysis showed the correlation between clinicopathological features and CSRS score. (b) Independent prognostic factors of OS were screened by Cox regression analysis. (c) Nomogram combining CSRS score and metastatic. (d) Nomogram assessed the calibration curves for OS survival at 1, 3, and 5 years. (e) Clinical decision-making benefits of the nomogram, CSRS score, and clinicopathologic feature. (f) ROC curve of CSRS score, nomogram, and clinicopathologic feature to predict 1–5-year survival.

The clinicopathological features and CSRS score of OS samples given in TARGET were included in univariate Cox regression analysis, and multivariate Cox regression analysis was performed to determine the independent prognostic factor CSRS score and metastatic of OS (Figures 8(a) and 8(b)). The selection of these two independent prognostic variables generated a nomogram, which showed that CSRS score had the greatest influence on the survival prediction of nomogram (Figure 8(c)). Nomogram's prediction curves for OS survival at 1, 3, and 5 years were close to the actual observational calibration curves (Figure 8(d)). DCA showed that nomogram and CSRS score had the greatest value in predicting clinical prognosis (Figure 8(e)). From the time-dependent ROC curve, the AUC predicted by CSRS score and nomogram for OS 1-, 3-, and 5-year survival were very close and much larger than that of metastatic, gender, or age (Figure 8(f)).

## 4. Discussion

CS is an essential cellular process in cancer. Previous studies have revealed the contribution of CS to the molecular classification of two cancers. Based on 17 genes prognostic of senescence, a study identified two distinct molecular types of colorectal cancer using ConsensusClusterPlus [23]. The other bioinformatics analysis of clear cell renal cell carcinoma (ccRCC) used unsupervised consensus clustering to identify three aging subtypes in ccRCC samples of TCGA based on GSVA enrichment scores of aging-related biological processes [24]. However, the identification and characterization of the cellular senescence subtypes of OS are still lacking [25]. In this study, 30 CS-related genes from CellAge divided OS into three CS-related subtypes, and the relationship between each of the 30 CS-related genes that define the three osteosarcoma subtypes has also not been reported. Of all the CS-related genes analyzed, 4 were identified to construct gene signature, which could predict the prognosis and clinical outcome of OS patients and reflect TME and treatment response.

Immunophenotypic analysis can be used as a very powerful tool to help better understand the complexity of immune response in osteosarcoma and the use of immunotherapy in this malignant tumor [26]. We analyzed the TIICs and TME of different CS subtypes and confirmed that CS showed the highest proportion of plasma cells and CD8 T cells and activated CD4 memory T cells and the highest stromal score, immune score, and comprehensive TME score. Plasma cells have been reported to be associated with good outcomes for a variety of cancers [27]. Similarly, high levels of CD8 T cells were associated with high survival rates in triple negative breast cancer [28]. Our analysis of the clinical results of three CS-related subtypes confirmed that C3 had the best prognosis, in addition to having the lowest probability of metastasis, and more than that, based on the expression of immune checkpoints in the three CS-associated subtypes and several scores calculated by TIDE, C3 seemed to be more likely to respond to ICB treatment.

In addition to providing insight into the CS-related subtypes, combining their analysis with biomarker identification may provide avenues for further cancer CS research, and ultimately improve the prognosis of patients [5]. In this study, four CS-related genes were constructed by using Lasso Cox algorithm, and the model was verified in 53 samples of GSE21257. Four genes in CSRS play a role in affecting cell senescence or malignant behavior of tumor through various mechanisms. The expression of MYC is significantly correlated with metastasis and poor prognosis of OS [29]. Signal transduction of super enhancer driven by MYC is an important mechanism of OS [30]. MYC activation induces the expression of p16 (INK4a) and p21 (Cip1) and resulted in Cdk2-deficient CS [31]. Inhibition of MYC reprograms TME by recruiting T lymphocytes and activating the CD40/CD40L system in osteosarcoma [32]. Overexpressed distal-less homeobox 2 (DLX2) is associated with adverse clinical outcomes in hepatocellular carcinoma [33] and gastric adenocarcinoma [34]. DLX2 reduces CS by regulating p53 function [35]. Eph receptor A3 (EPHA3) plays a tumor suppressive role in esophageal squamous cell carcinoma [36], and the loss of its expression is related to lymph node metastasis and TNM staging of colorectal cancer [37]. This molecule regulates multidrug resistance in lung cancer through the PI3K/BMX/STAT3 signaling pathway [38]. LIM kinase 1 (LIMK1) plays a key role in multidrug resistance of OS [39]. However, there is no publication elucidating the application of these four genes to CSRS in OS.

The CSRS we developed is of great significance for the determination of clinical results in patients with OS. On the one hand, CSRS could predict the prognosis of patients with different clinical features. The survival time of samples with high CSRS score was significantly shorter and the survival rate was significantly lower, so high-risk patients should receive more frequent clinical monitoring to prevent the deterioration of OS. On the other hand, we also detected a significant correlation between CSRS score and a variety of known predictors of ICB treatment (the expression of immune checkpoints and several types of TIDE score). What should be of concern is that CSRS score could also be used to screen patients suitable for doxorubicin treatment.

There are still several limitations in our research. First of all, the results of this study were obtained only through bioinformatics analysis of two common datasets and need to be verified from a multicenter queue. Prospective clinical trials are also necessary. Secondly, the regulatory mechanism of CSRS in OS has not been studied in detail and needs to be explored by in vitro cell and in vivo solid tumor experiments.

## 5. Conclusions

In summary, our study identified a molecular classification determined by CS-related genes, dividing OS into three CS-related subtypes with unique clinical outcomes and TME status. A CSRS score model was developed to provide potential indicators for clinical prognosis and immunotherapy in patients with OS.

## Data Availability

The dataset analyzed in this study could be found at (https://www.ncbi.nlm.nih.gov/geo/query/acc.cgi?acc=GSE21257), and (https://ocg.cancer.gov/programs/target).

## Supplementary Materials

Supplementary Materials
*Figure S1.* Flow pictures of the overall work of this study. *Figure S2.* Clinical characteristics of three CS-related subtype manifestations of OS. (A) The clinicopathological features of three OS subtypes in TARGET dataset. (B) The clinical characteristics of three CS-related subtypes in GSE21257 dataset. *Figure S3*. Pathways significantly activated and suppressed in C1 relative to C3. (A) In the TARGGE dataset, pathways that were significantly activated and suppressed relative to C3. (B) The normalized enrichment score of C1 relative to C3 in the two data sets of OS. *Figure S4.* Immunotherapy response or drug sensitivity assessment for three CS-related subtypes in GSE39055. (A) Survival curves of three CS-related subtypes in the GSE39055 dataset. (B) The expression of immune checkpoints in three CS-related subtypes of GSE39055. (C) The IC50 of cisplatin, doxorubicin, methotrexate, and paclitaxel in three OS molecular subtypes of GSE39055. *Figure S5.* Verification of CSRS in GSE21257 dataset. (A) Survival curves of different CSRS samples in GSE21257 dataset. (B) Accuracy evaluation of CSRS in GSE21257 dataset.
