# Supplementary figures and images for: Subtype Classification and Prognosis Signature Construction of Osteosarcoma Based on Cellular Senescence-Related Genes
Source: J Oncol. 2022 Sep 5;2022:4421952. doi: 10.1155/2022/4421952 (PMC9467774; doi:10.1155/2022/4421952)

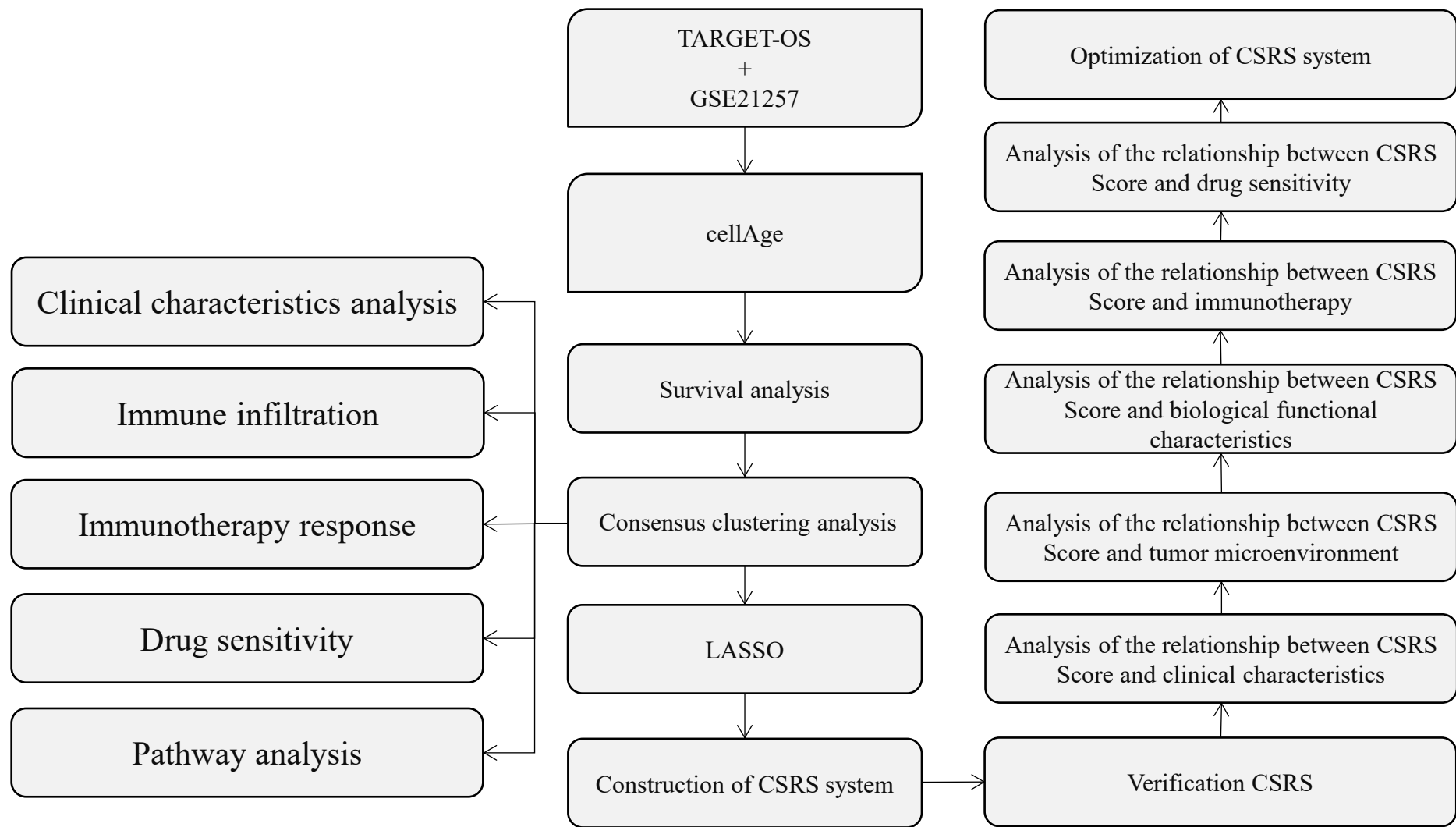

Supplement: Supplementary Materials — Figure S1. Flow pictures of the overall work of this study. Figure S2. Clinical characteristics of three CS-related subtype manifestations of OS. (A) The clinicopathological features of three OS subtypes in TARGET dataset. (B) The clinical characteristics of three CS-related subtypes in GSE21257 dataset. Figure S3. Pathways significantly activated and suppressed in C1 relative to C3. (A) In the TARGGE dataset, pathways that were significantly activated and suppressed relative to C3. (B) The normalized enrichment score of C1 relative to C3 in the two data sets of OS. Figure S4. Immunotherapy response or drug sensitivity assessment for three CS-related subtypes in GSE39055. (A) Survival curves of three CS-related subtypes in the GSE39055 dataset. (B) The expression of immune checkpoints in three CS-related subtypes of GSE39055. (C) The IC50 of cisplatin, doxorubicin, methotrexate, and paclitaxel in three OS molecular subtypes of GSE39055. Figure S5. Verification of CSRS in GSE21257 dataset. (A) Survival curves of different CSRS samples in GSE21257 dataset. (B) Accuracy evaluation of CSRS in GSE21257 dataset. [file 4421952.f1.zip › 4421952.f1/Figure S1 (2).pdf]

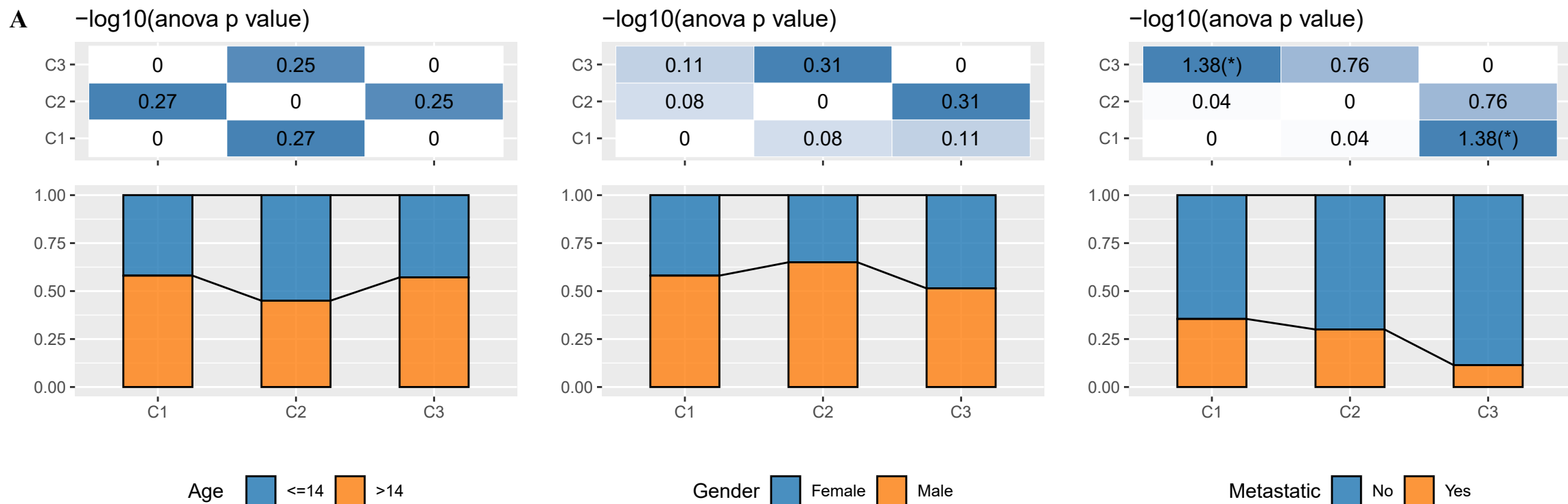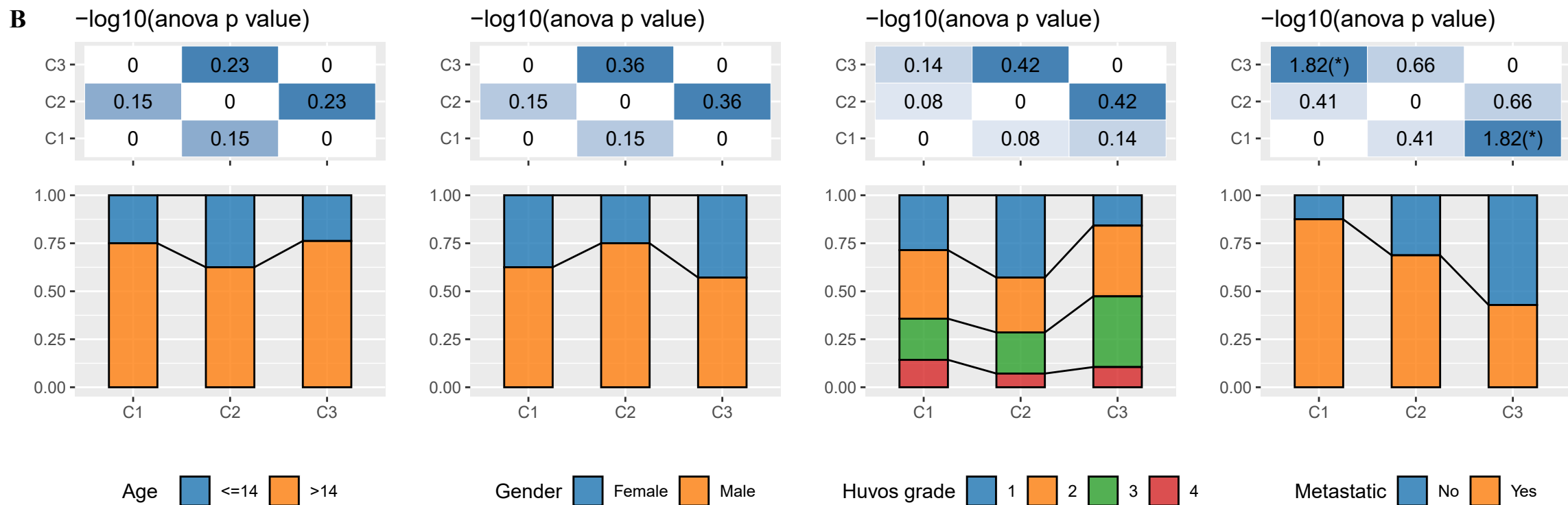

Supplement: Supplementary Materials — Figure S1. Flow pictures of the overall work of this study. Figure S2. Clinical characteristics of three CS-related subtype manifestations of OS. (A) The clinicopathological features of three OS subtypes in TARGET dataset. (B) The clinical characteristics of three CS-related subtypes in GSE21257 dataset. Figure S3. Pathways significantly activated and suppressed in C1 relative to C3. (A) In the TARGGE dataset, pathways that were significantly activated and suppressed relative to C3. (B) The normalized enrichment score of C1 relative to C3 in the two data sets of OS. Figure S4. Immunotherapy response or drug sensitivity assessment for three CS-related subtypes in GSE39055. (A) Survival curves of three CS-related subtypes in the GSE39055 dataset. (B) The expression of immune checkpoints in three CS-related subtypes of GSE39055. (C) The IC50 of cisplatin, doxorubicin, methotrexate, and paclitaxel in three OS molecular subtypes of GSE39055. Figure S5. Verification of CSRS in GSE21257 dataset. (A) Survival curves of different CSRS samples in GSE21257 dataset. (B) Accuracy evaluation of CSRS in GSE21257 dataset. [file 4421952.f1.zip › 4421952.f1/Figure S2 (2).pdf]

A

TARGET C1 vs C3

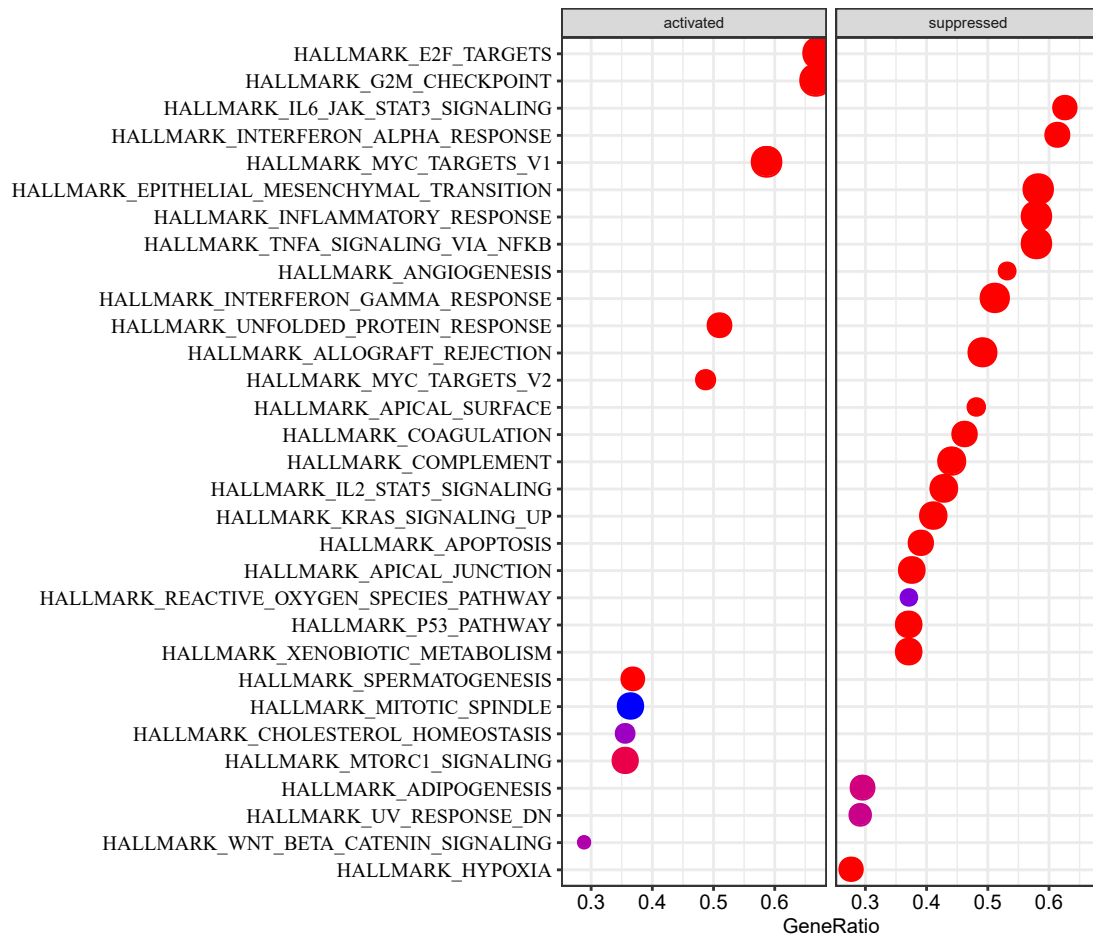

B

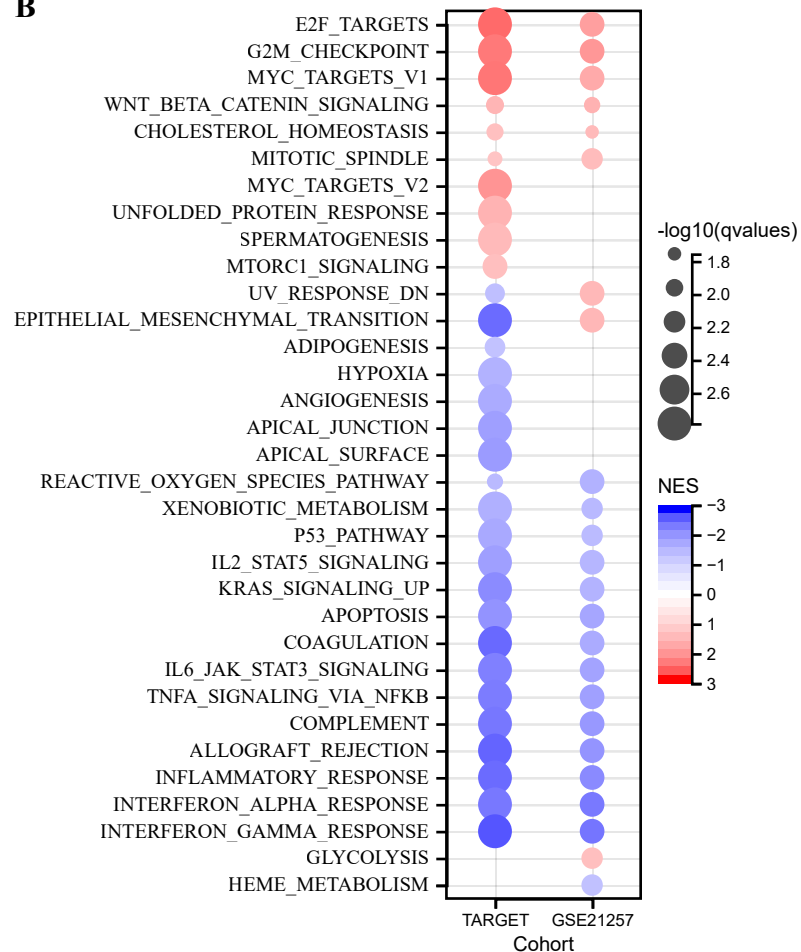

Supplement: Supplementary Materials — Figure S1. Flow pictures of the overall work of this study. Figure S2. Clinical characteristics of three CS-related subtype manifestations of OS. (A) The clinicopathological features of three OS subtypes in TARGET dataset. (B) The clinical characteristics of three CS-related subtypes in GSE21257 dataset. Figure S3. Pathways significantly activated and suppressed in C1 relative to C3. (A) In the TARGGE dataset, pathways that were significantly activated and suppressed relative to C3. (B) The normalized enrichment score of C1 relative to C3 in the two data sets of OS. Figure S4. Immunotherapy response or drug sensitivity assessment for three CS-related subtypes in GSE39055. (A) Survival curves of three CS-related subtypes in the GSE39055 dataset. (B) The expression of immune checkpoints in three CS-related subtypes of GSE39055. (C) The IC50 of cisplatin, doxorubicin, methotrexate, and paclitaxel in three OS molecular subtypes of GSE39055. Figure S5. Verification of CSRS in GSE21257 dataset. (A) Survival curves of different CSRS samples in GSE21257 dataset. (B) Accuracy evaluation of CSRS in GSE21257 dataset. [file 4421952.f1.zip › 4421952.f1/Figure S3 (2).pdf]

**A**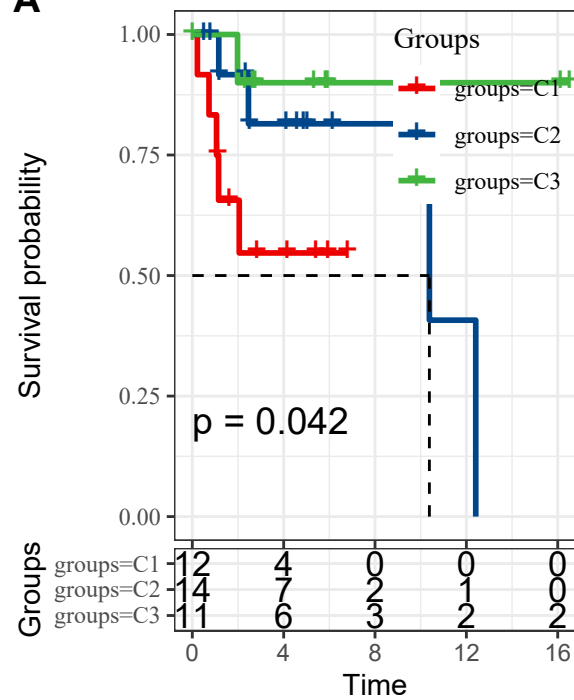**B**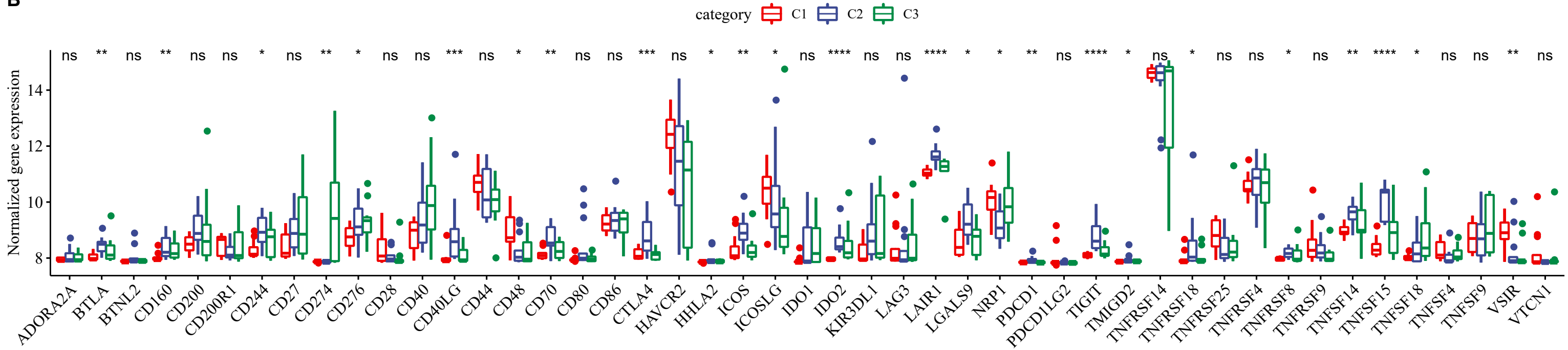**C**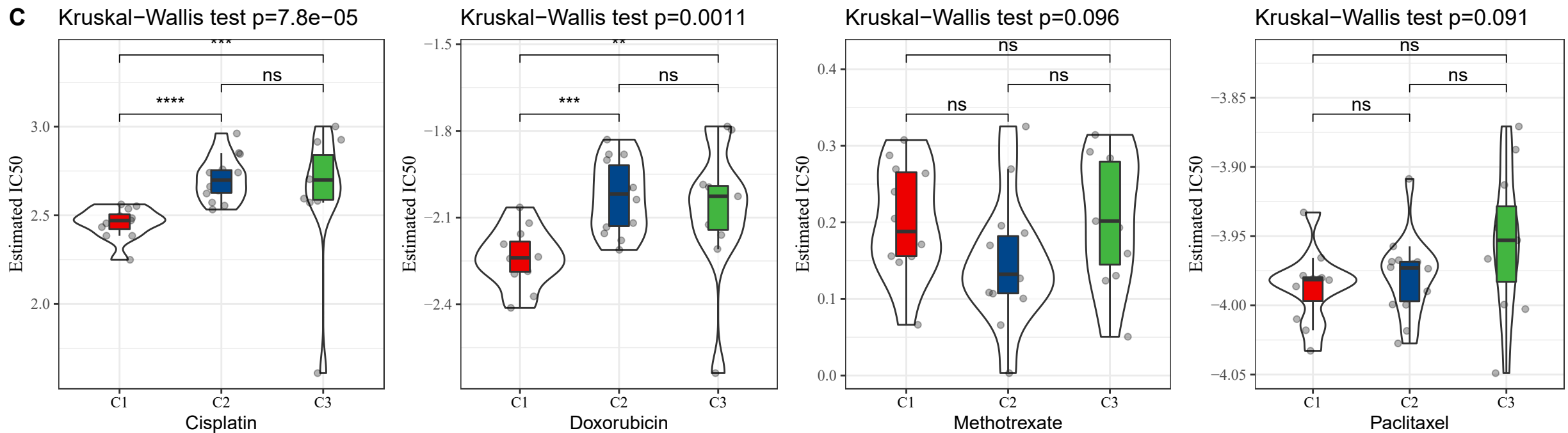

Supplement: Supplementary Materials — Figure S1. Flow pictures of the overall work of this study. Figure S2. Clinical characteristics of three CS-related subtype manifestations of OS. (A) The clinicopathological features of three OS subtypes in TARGET dataset. (B) The clinical characteristics of three CS-related subtypes in GSE21257 dataset. Figure S3. Pathways significantly activated and suppressed in C1 relative to C3. (A) In the TARGGE dataset, pathways that were significantly activated and suppressed relative to C3. (B) The normalized enrichment score of C1 relative to C3 in the two data sets of OS. Figure S4. Immunotherapy response or drug sensitivity assessment for three CS-related subtypes in GSE39055. (A) Survival curves of three CS-related subtypes in the GSE39055 dataset. (B) The expression of immune checkpoints in three CS-related subtypes of GSE39055. (C) The IC50 of cisplatin, doxorubicin, methotrexate, and paclitaxel in three OS molecular subtypes of GSE39055. Figure S5. Verification of CSRS in GSE21257 dataset. (A) Survival curves of different CSRS samples in GSE21257 dataset. (B) Accuracy evaluation of CSRS in GSE21257 dataset. [file 4421952.f1.zip › 4421952.f1/Figure S4 (1).pdf]

D

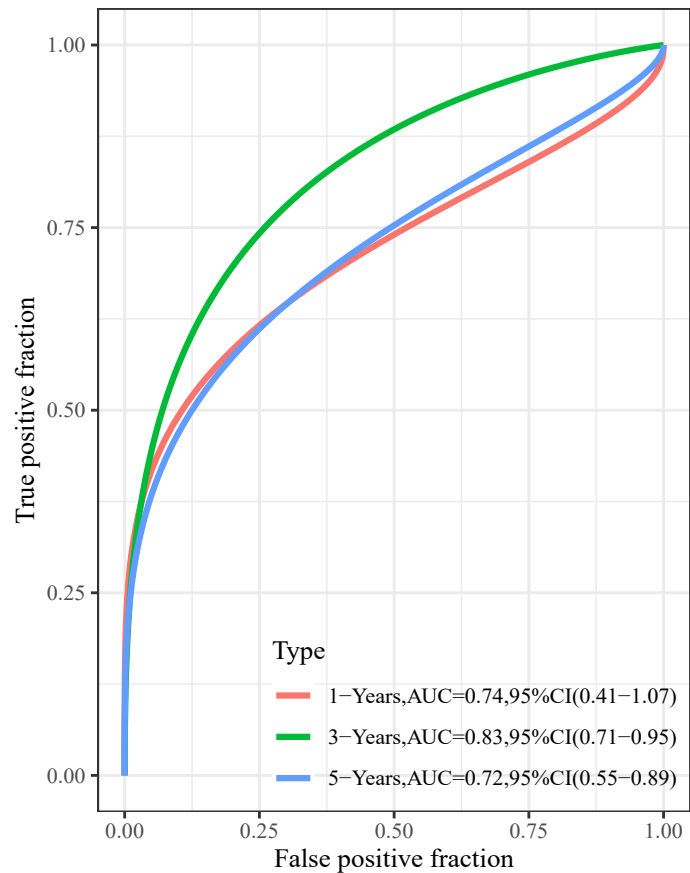

E

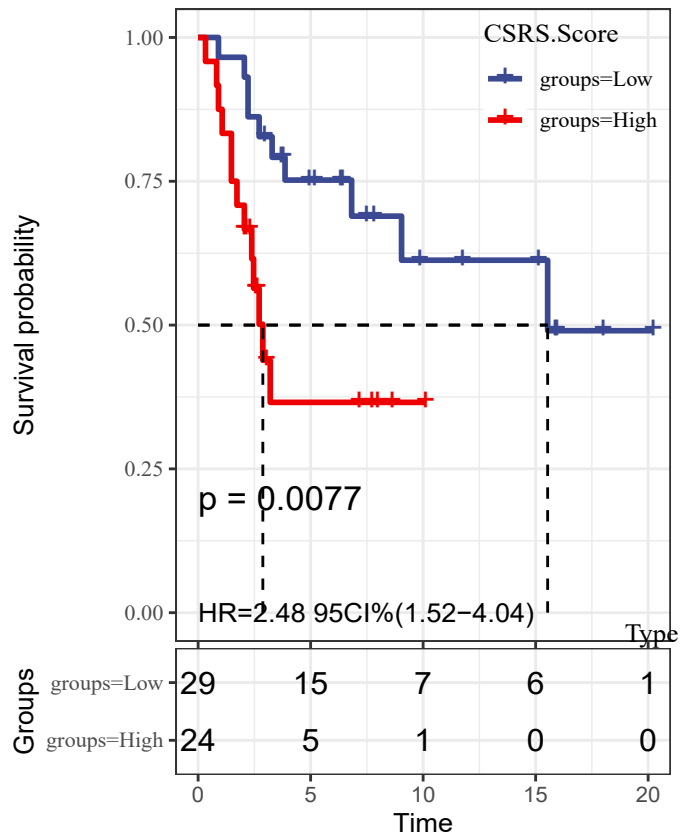

Supplement: Supplementary Materials — Figure S1. Flow pictures of the overall work of this study. Figure S2. Clinical characteristics of three CS-related subtype manifestations of OS. (A) The clinicopathological features of three OS subtypes in TARGET dataset. (B) The clinical characteristics of three CS-related subtypes in GSE21257 dataset. Figure S3. Pathways significantly activated and suppressed in C1 relative to C3. (A) In the TARGGE dataset, pathways that were significantly activated and suppressed relative to C3. (B) The normalized enrichment score of C1 relative to C3 in the two data sets of OS. Figure S4. Immunotherapy response or drug sensitivity assessment for three CS-related subtypes in GSE39055. (A) Survival curves of three CS-related subtypes in the GSE39055 dataset. (B) The expression of immune checkpoints in three CS-related subtypes of GSE39055. (C) The IC50 of cisplatin, doxorubicin, methotrexate, and paclitaxel in three OS molecular subtypes of GSE39055. Figure S5. Verification of CSRS in GSE21257 dataset. (A) Survival curves of different CSRS samples in GSE21257 dataset. (B) Accuracy evaluation of CSRS in GSE21257 dataset. [file 4421952.f1.zip › 4421952.f1/Figure S5 (1).pdf]
